# Evaluation of antibody response to BNT162b2 mRNA COVID-19 vaccine in patients affected by immune-mediated inflammatory diseases up to 5 months after vaccination

**DOI:** 10.1007/s10238-021-00771-3

**Published:** 2021-11-05

**Authors:** Davide Firinu, Andrea Perra, Marcello Campagna, Roberto Littera, Giuseppe Fenu, Federico Meloni, Selene Cipri, Francesca Sedda, Maria Conti, Michela Miglianti, Giulia Costanzo, Marta Secci, Gianmario Usai, Mauro Giovanni Carta, Riccardo Cappai, Germano Orrù, Stefano Del Giacco, Ferdinando Coghe, Luchino Chessa

**Affiliations:** 1 Department of Medical Sciences and Public Health, Policlinico Universitario – AOU di Cagliari, University of Cagliari, Azienda Ospedaliero Universitaria, SS 554-Bivio Sestu, 09042 Monserrato, Cagliari, CA Italy; 2 Department of Biomedical Sciences, University of Cagliari, Cagliari, Italy; 3 Associazione per l’Avanzamento della Ricerca per i Trapianti O.d.V., Non Profit Organisation, Cagliari, Italy; 4 Medical Genetics, Department of Medical Sciences and Public Health, University of Cagliari, Cagliari, Italy; 5 Department of Neuroscience, A.O. Brotzu, Cagliari, Italy; 6 Laboratory Clinical Chemical Analysis and Microbiology, University Hospital of Cagliari, Cagliari, Italy

**Keywords:** Covid-19, Vaccination, Immunogenicity, Autoimmune disease, IMID, BNT162b2

## Abstract

SARS-CoV-2 vaccination with mRNA product BNT162b2 elicited high immunogenicity in healthy subjects in trials. This study aims to better understand the factors that influence the humoral immune response to vaccination against SARS-CoV-2 in patients with immune-mediated inflammatory diseases (IMIDs). We enrolled patients and healthy healthcare workers control group (HCW) that underwent mRNA BNT162b2 vaccination and measured the serum IgG anti-S-RBD response at booster dose (T1), one month after booster dose (T2) and up to 5 months (T3). Demographic, disease-specific and vaccination data were recorded. Vaccination response of 551 participants naïve to SARS-CoV-2 infection were included in HCW and 102 in the IMID group, analyzing separately those on anti-CD20. At T2 all naïve HCW developed anti-S-RBD-IgG, while 94% of IMID responded (*p* < 0.001). IMID patients had a significantly different level of IgG than HCW at both T1 (*p* = 0.031), T2 (*p* < 0.001), while there was no significant difference at T3. There were no statistically significant differences according to the IMID type or to ongoing treatment with immunosuppressants, corticosteroids or biological drugs other than anti-CD20. The proportion and magnitude of response was significantly lower in IMID treated with anti-CD20 drugs. There was a correlation with age at T1 and at T2 but not at T3, stronger in patients than in HCW. Immune response close after BNT162b2 vaccination is reduced in patients with IMID, but there is no significant difference at 5 months. The measured reduction is related to age and the disease itself rather than treatments, with the exception of anti-CD20 drugs.

**Supplementary Information:**

The online version contains supplementary material available at 10.1007/s10238-021-00771-3.

## Introduction

The extent of the profound immunological and non-immunological responses linked to SARS-CoV-2 infection is currently being investigated worldwide, due to the huge death-toll of SARS-CoV-2 pandemic and the short-term consequences of COVID-19. The first SARS-CoV-2 vaccines are among the most remarkable science and medicine accomplishments in modern history and offer realistic hope for an end to the COVID-19 pandemic.

Epidemiological models to estimate the magnitude and timing of future COVID-19 cases, given different assumptions regarding the protective efficacy and duration of the adaptive immune response to SARS-CoV-2, range widely from sustained epidemics to near elimination [1]. Even in the best-case scenario of an apparent elimination, a resurgence in contagion could be possible as late as 2024 [2]. Thus, to alleviate the direct consequences of the ongoing pandemic detailed and reliable data on the epidemiology, natural history and treatment possibilities of SARS-CoV-2 infection, as well as about vaccine response are needed. Vaccination represents the only reliable means to quickly mitigate the spread and impact of COVID-19 in the forthcoming period. Assessment and post-vaccine monitoring of anti-SARS-CoV-2 antibody specifically targeting and thereby inactivating the spike protein and/or its receptor binding domain (RBD) may inform about the humoral response and its duration [3].

The Pfizer-BioNTech COVID-19 (BNT162b2) vaccine consisting of a lipid nanoparticle-formulated mRNA vaccine encoding the prefusion spike glycoprotein of SARS-CoV-2 received the conditional marketing authorization from the European Medicine Agency (EMA) on December 21, 2020 and was approved by the Food and Drug Administration (FDA) on January 14, 2021. The overall immune response to BNT162b2 has been reported [4], inducing SARS-CoV-2 specific spike-protein (and/or its RBD) B-cells and neutralizing antibody response and generation of specific polyfunctional CD8 + and CD4 + T-cells. Although the overall immune effects of the BNT162b2 vaccine has been reported, the profile of humoral immune profile remain to be further investigated in selected subgroups such as immune-mediated inflammatory diseases (IMID) [5].

Since infections are a relevant cause of morbidity and mortality in patients with IMID [6], it is relevant to address this question because the worldwide spread of the SARS-CoV-2 infection forces a rapid vaccination of patients suffering from these diseases.

Moreover, in these patients the infection risk may be even higher for both the altered regulation of the immune system itself and for the immunosuppressive effects of medications [7, 8].

Indeed, vaccinations in this population are complicated by disease-modifying immunosuppressive agents or antirheumatic drugs (either conventional, targeted synthetic or biologicals), which modulate or suppress key targets of the immune system and potentially decrease the immunogenicity and efficacy of the vaccines [9].

However, there is scant available data on real-world cohorts of vaccinated subjects and about response to COVID-19 mRNA vaccines in patients affected by IMID [10–14], in particular beyond 4–8 weeks after the full vaccination course. In a prospective observational study focused on COVID-19 infection and vaccination (CORIMUN study), we tested the antibody response to SARS-CoV-2 spike protein over 5 months after vaccination. This study aimed to describe the characteristics of humoral response induced by mRNA-based vaccine BNT162b2 [15] in subjects affected by IMID and analyze the impact of treatments.

## Methods

### Patient selection

We enrolled consecutive subjects aged > 18 years, having received COVID-19 vaccine Pfizer/BioNTech BNT162b2 (two doses 21-days apart of 30 μg mRNA vaccine Comirnaty by Pfizer Inc, NY, USA) and deliberately given their Informed Consent to participate in the study. Pregnancy, transplantation, known immunodeficiency or lymphoproliferative disorders were exclusion criteria. Among enrolled subjects, we defined two groups:

- Vaccinated subjects at our hospital with concurrent IMID, grouped as: (1) ankylosing spondylitis (AS), psoriasis, psoriatic arthritis (PsA); (2) rheumatoid arthritis (RA) (3) systemic lupus erythematosus; (4) miscellaneous systemic disorders (5) inflammatory bowel disease (6) multiple sclerosis;
- A control group of well-characterized subjects enrolled among healthy healthcare workers (HCW) enrolled at our hospital, without any of the immune-mediated diseases listed above, no evidence of immunodeficiency or taking relevant medications.

To ascertain prior infection with SARS-CoV-2, subjects were asked if they had a positive PCR test in the past and were cross-matched with the database of positive rt-PCR tests at the laboratory and hospital records. We also checked data of serological testing for health surveillance in HCW from June to December 2020 of both IgM and IgG antibodies with the 2019-nCov (Snibe, Shenzhen, China) chemiluminescent analytical system (CLIA) assay on MAGLUMI platform that detects antibodies of natural infection to SARS-CoV-2 Spike-(S) protein and N-protein with high sensitivity and specificity.

Each group included both naïve and previously infected (rt-PCR confirmed infection) subjects. Naïve subjects had not been previously infected by SARS-CoV-2 (testing since April 2020 gave repeatedly negative rt-PCR test for SARS-CoV-2; repeatedly undetectable IgG or IgM antibodies, also at the end of December 2020, before vaccination).

### Sample collection and storing

10 mL of peripheral blood was obtained by venipuncture immediately before each vaccine dose and defined as T0, before first dose; T1, at second vaccine shot (+ 21 days from T0); T2 at day 51 (T1 + 28 days); T3 at day 151 after the first vaccine dose. The serum was separated by centrifugation (2000 × g for 15 min) within 3 h of collection and aliquots were stored at − 80 °C until use.

### Serological studies

The antibody response induced by vaccine to S protein (primary endpoint) was detected with the anti-SARS-CoV-2 S-RBD IgG (Snibe Diagnostics, New Industries Biomedical Engineering Co., Ltd, Shenzhen, China) on a MAGLUMI analyzer, to titrate levels of specific IgG at specific timepoints. Analytical and clinical features of the assay, including the correlation with neutralization by using plaque reduction neutralization test (PRNT) 50 titer has been previously investigated [16].

### Statistical analysis

Patient characteristics were summarized using means, medians, standard deviations, ranges and percentages as appropriate. Chi-squared tests of independence and Fischer’s exact tests were used for categorical data. Mann–Whitney *U* and Kruskal–Wallis tests were used for unpaired continuous data. Linear regression was used to evaluate the relationship between the dependent variable (antibody titer) and the clinical and demographic characteristics of patients as independent variables. All reported p-values represent 2-tailed tests, with *p* ≤ 0.05 considered statistically significant. All variables were analyzed using SPSS.

### Ethical aspects

Patients were recruited and enrolled in the study protocol at the University Hospital of Cagliari. Written informed consent was obtained from all patients and controls in accordance with the ethical standards (institutional and national) of the local human research committee. The study protocol, including informed consent procedures, conforms to the ethical guidelines of the Declaration of Helsinki and was approved by the responsible ethics committee (Ethics Committee of the Cagliari University Hospital approval May 27, 2020; protocol number GT/2020/10894 and extension approved January 27, 2021). Records of written informed consent are kept on file and are included in the clinical record of each patient.

## Results

Among subjects vaccinated and naïve to SARS-CoV-2 Infection, 551 participants were included in the control group (HCW), and 102 were in the IMID group. Vaccinated HCW with previous SARS-CoV-2 infection were 111, and 9 in the IMID group. Baseline characteristics of both groups are shown in Table 1. Mean study dropout rate at T3 versus T2 was of 15%.

**Table 1:** Characteristics of patients and controls

Subjects characteristics	HCW, SARS-CoV-2 naive (*n* = 551)	IMID, SARS-CoV-2 naive (*n* = 95)	IMID treated with anti-CD20 naive (*n* = 7)	HCW, previous SARS-CoV-2 (*n* = 111)	IMID previous SARS-CoV-2 (*n* = 9)
Age (years), median	51 (39–58)	56 (42.5–66)	58 (50–69.25)	48 (35–55)	50 (30.5–61.5)
Males, %	31.8	27.5	14.3	38.7	33.3
BMI (IQR)	23.18 (20.79–25.95)	23.56 (21.4–26.6)	21.77 (19.9–23.7)	23.93 (21.66–26.32)	22.65 (20.47–25.53)
Current smoker, %	16.6	16.9	14.3	11.5	11
Diabetes, %	3.3	6.4	14.3	3.8	11.1
*IMID type*					
SLE	0	19 (23.5)	2 (28.6)	0	1 (11.1)
RA	0	19 (23.5)	1 (14.3)	0	2 (22.2)
PsA, Psoriasis, AS	0	20 (24.7%)		0	1 (11.1)
Miscellaneous systemic disorders^a^	0	16 (19.8)	2 (28.6)	0	1 (11.1)
IBD	0	4 (4.9)		0	2 (22.2)
Multiple sclerosis	0	3 (3.7)	2 (28.6)	0	2 (22.2)
*Immunomodulatory treatment*					
Glucocorticoids, *n* (%)	0	43 (53.1)	5 (71.4)	0	2 (22.2)
Prednisone eq/day, mg	0	5 (5–8.75)	5 (5–10)	0	3.75 (2.5–3.75)
Glucocorticoid and/or any immunosuppressive drug, *n* (%)	0 (0)	70 (74.5)	6 (87.5)	0 (0)	5 (55.6)
Anti-TNF-α *n* (%)	0 (0)	11 (13.6)	0 (0)	0 (0)	1 (11.1)
Non-anti-TNF biologicals (ustekinumab, secukinumab, abatacept, tocilizumab and vedolizumab) *n* (%)	0 (0)	10 (12.3)	0 (0)	0 (0)	0 (0)
csDMARDs^b^ *n* (%)	0 (0)	41 (51.2)	3 (42.9)	0 (0)	1 (11.1)
Other drugs^c^ *n* (%)	0 (0)	5 (6.3)	0 (0)	0 (0)	1 (11.1)

^a^Sjögren syndrome, Behçet’s disease, IgA nephropathy, IgG4-related disease, Eosinophilic granulomatosis with polyangiitis, giant-cell arteritis, autoimmune hepatitis and UCTD

^b^csDMARDs: methotrexate, sulfasalazine, leflunomide, hydroxychloroquine, mycophenolate, azathioprine and cyclosporin

^c^Interferon, colchicine, JAK-inhibitors, belimumab and dimethyl fumarate

Both cohorts included only Caucasian participants, and patients continued their treatment before or after the two vaccine doses, without stopping or tapering their drug(s), only avoiding the administration of injective drugs on ± 3 days of the vaccine date.

Due to the well-known impairment of immunogenicity linked to anti-CD20 agents [17], patients taking this medication were analyzed separately.

There was no difference in age, gender, BMI and cigarette smoking comparing these groups (Kruskall–Wallis χ2, *p* > 0.05).

### Immunogenicity of BNT162b2 in SARS-CoV-2 naïve patients with IMID and controls

All subjects in the naive HCW group developed a positive antibody response (defined as > 1 AU/ml or higher) at T1 (364/364) and T2 (551/551) as compared with 90% (27/30) at T1 and 94% (63/67) at T2 in the IMID group. A statistically significant difference was found at T1 (*p* = 0.002) and T2 (*p* < 0.001; Fisher’s exact test).

At T1, the median IgG anti-S-RBD levels in the IMID group were 14.08 AU/mL (IQR, 5.08–27.8), increasing to 146.39 AU/mL (IQR, 53.93–295) at T2 and then decreasing to 60.65 AU/mL (IQR, 21.1–96,55) at T3 (− 59% vs. T2).

In HCW controls, at T1 the median IgG anti-S-RBD levels were 23.66 AU/mL (IQR, 9.76–47.9), increasing to 217 AU/mL (IQR, 134.8–389.9) at T2 and then decreasing to 46.76 AU/mL (IQR, 26.26–81.57) at T3 (− 79% vs. T2).

**Fig. 1:**
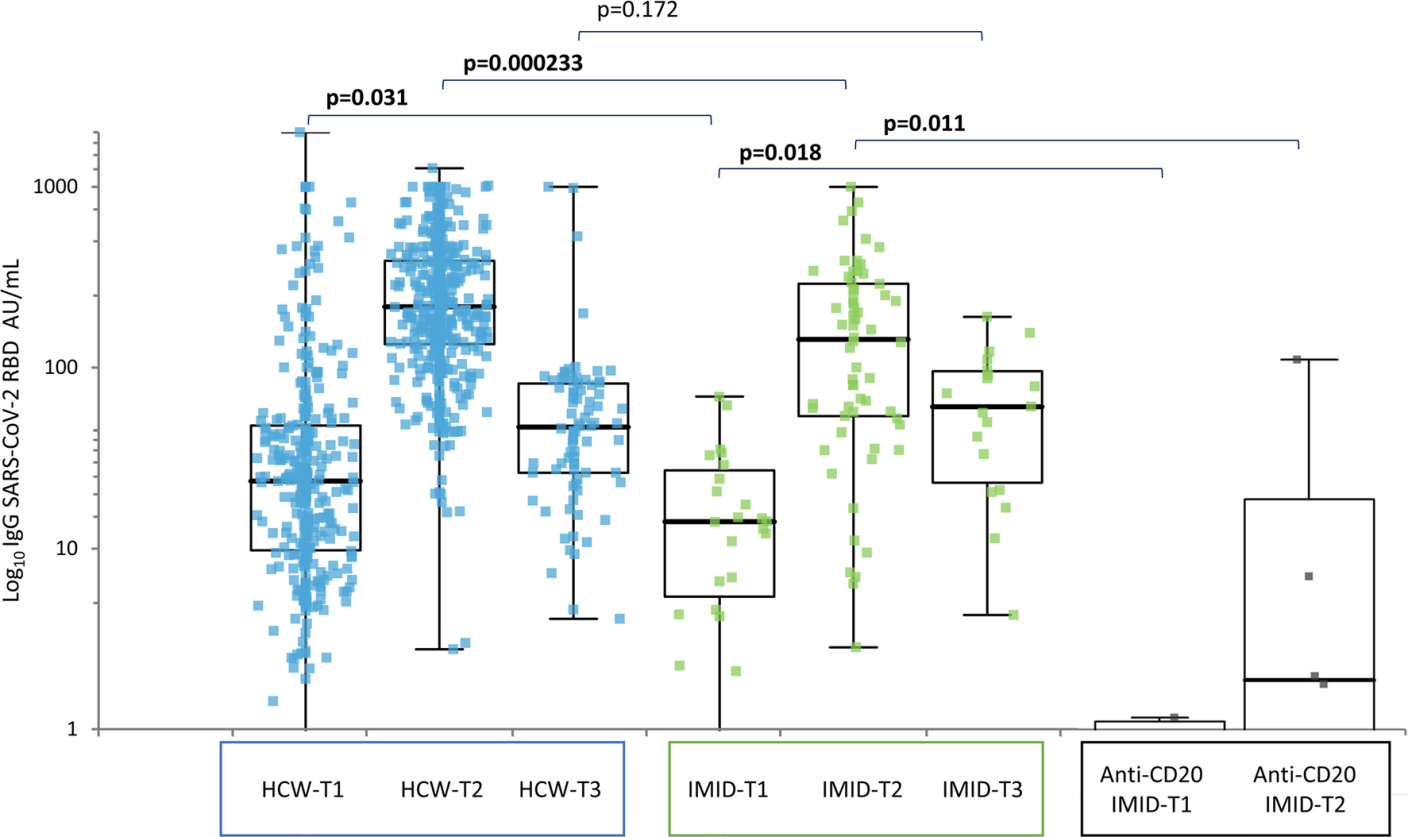
SARS-CoV-2 anti-S-RBD IgG immune responses in naïve patients and control subjects. IgG titer anti-S-RBD IgG (AU/L) elicited by BNT162b2 at T1 (booster dose), T2 (28 days after booster) and T3 (151 days after first dose) among healthy healthcare workers (HCW) and IMID subjects and IMID treated with anti-CD20 agents (anti-CD20 IMID), naïve to SARS-CoV-2 infection. All subjects were vaccinated with two doses, 21 days apart. Comparison between groups by Mann–Whitney test, bold indicates statistical significance

The median IgG anti-S-RBD levels in the IMID group were significantly lower than control group test at T1 (*p* = 0.031) and T2 (*p* = 0,000,233), while there was no significant difference at T3 (*p* = 0.172) (Fig. 1).

#### IMID subgroups and antibody levels

There were no statistically significant differences between subgroups of IMID enrolled in the study for age, BMI and proportion of patients currently treated with immunosuppressive treatment (Kruskal–Wallis test).

**Fig. 2:**
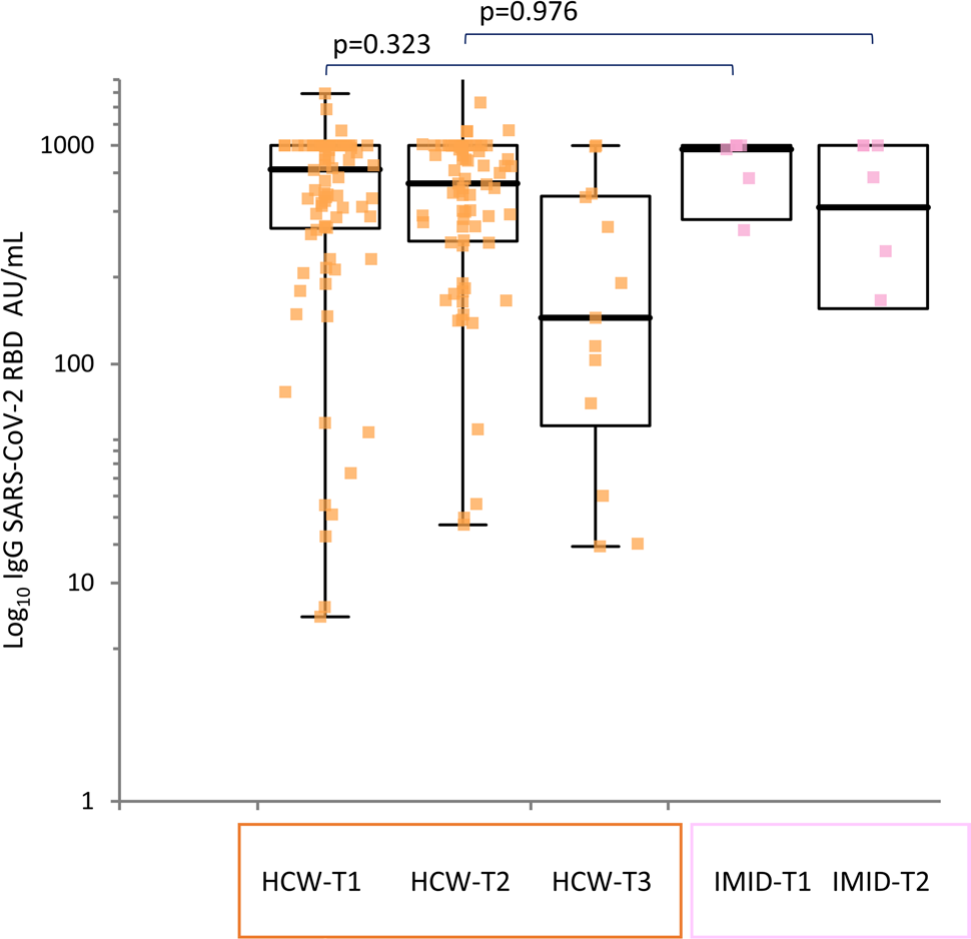
SARS-CoV-2 anti-S-RBD IgG immune responses in patients and control subjects with previous SARS-CoV-2. IgG titer Anti-S-RBD IgG (AU/L) elicited by BNT162b2 at T1 (booster dose), T2 (28 days after booster) and T3 (151 days after first dose) among healthy healthcare workers (HCW) and IMID subjects with previous SARS-CoV-2 infection. All subjects were vaccinated with two doses, 21 days apart. Comparison between groups by Mann–Whitney test, bold indicates statistical significance

The rate of seropositivity at T2 was similar among the subgroups of diseases included in the study and there were no statistically significant differences between subgroups of subjects at T1, T2 and T3 (*p* > 0.05, Kruskal–Wallis test).

The median IgG anti-S-RBD levels and IQR at T1 and T2 for each IMID subgroup are presented in the supplementary materials, Table S1.

#### Correlation of treatments and antibody levels

Comparing the response to vaccination in patients treated with GC, GC and/or immunosuppressive drug we did not find a significant difference in IgG anti-S-RBD levels at T1, T2 and T3 (*p* > 0,05). Similarly, we found no differences for those under anti-TNF-alpha drugs (median of those treated at T2 139.7 AU/mL; IQR, 61–279) for the subgroups of RA and PsA/Psoriasis, or for those treated with biologicals different from TNF-alpha inhibitors (ustekinumab, secukinumab, abatacept, tocilizumab and vedolizumab) (*p* > 0,05), csDMARDs (supplementary materials, Table S2), or for other drugs (interferon, colchicine, Jak-inhibitors, belimumab and dimethyl fumarate); (for all at T1, T2 T3; comparison drug vs. no drug).

#### Correlation with subjects’ characteristics

Among the demographic characteristics, age could influence the response to vaccination in both patients and healthy controls. In fact, the Spearman test showed a correlation between age and antibody response at T1 and at T2. This correlation is stronger in patients than in healthy controls (T1: rho: − 0.24 in HCW vs. − 0.41 in patients). At T3, there was no correlation between age and antibody response (supplementary materials, Tables S4 and S5).

Linear regression models were tested including IgG anti-RBD titer at each timepoint (T1, T2 and T3) as dependent variable and age, gender, glucocorticoid, treatment with immunosuppressant, treatment with biologicals, disease duration as independent variables. No significant relationship between IgG titer at T1, T2 and T3 and multiple combinations of clinical and demographic variables was detected except for age (supplementary materials, Table S6).

### Immunogenicity in the group of naïve IMID patients treated with anti-CD20 drugs

At T2, 2/7 (28.6%) subjects were non-responders in the IMID subjects previously treated with B-cell depleting agents (rituximab or ocrelizumab) after two doses of BNT162b2. They were non-responders also at T1. The median time from last drug infusion until vaccination was 5.5 months (IQR 5.5–6). The median IgG anti-S-RBD levels in this group were significantly lower than other patients with IMID naive to anti-CD20 at both T1 (*p* = 0.018) and T2 (*p* = 0.011) (supplementary materials, Table S3).

### Immunogenicity of BNT162B2 in IMID and controls with previous SARS-CoV-2 infection

In the HCW group 100% (111/111) developed a positive antibody response (defined as > 1 AU/ml or higher) and an increase in IgG titer at T1 as compared with 88.8% (8/9) of responders at both T1 and T2 in the IMID group (Fig.2 ).

At T1, the median IgG anti-S-RBD levels in the IMID group were 980.2 AU/mL (IQR, 632–1000), then 714.9 AU/mL (IQR, 58.31–292.35) at T2.

In controls, at T1 the median IgG anti-S-RBD levels were 778.2 AU/mL (IQR, 416–1000), 670 AU/mL (IQR, 363.15–1000) at T2 and then decreasing to 162 AU/mL (IQR, 45.44–590) at T3.

The median IgG anti-S-RBD levels in the IMID group with previous SARS-CoV-2 were not significantly different than HCW group at T1 (*p* = 0.323) and T2 (*p* = 0.976).

### Tolerability of BNT162b2 in patients with IMID

In general, vaccination was well tolerated in all patients and controls. No relapse or overshooting inflammatory response to vaccination was observed in patients with IMID. We detected no relevant safety issues in the enrolled subjects of both groups. Side effects were generally more frequent after the booster dose, and pain at the injection site was most frequently observed in both groups without statistically significant differences between IMID and HCW (both naïve and non-naïve) and among IMID subgroups. Systemic side effects (injection site reaction, headache, chills and arthralgia) were less frequent in patients than in controls (23.2% vs. 40%, this finding being significant among naïve IMID (*p* = 0.04, no differences between disease subgroups) and associated with ongoing glucocorticoids (*p* = 0.032).

## Discussion

The ongoing COVID-19 pandemic is being limited also by an unprecedented vaccine program with the innovative use of mRNA vaccines [8]. Due to limitations in the mRNA vaccine trials and to prioritization given to IMID patients for vaccination data about response to COVID-19 mRNA vaccines are of utmost importance to provide data about immunogenicity in this population and to inform health policies. This study shows that BNT162b2, based on mRNA technology, is highly immunogenic in IMID patients as the vast majority (94%) are responders (defined by development of IgG to S-RBD ≥ 1 AU/ml) one month after the second dose. In the subgroup of patients under B-cell depleting agents, this proportion is reduced to about 30%. Among naïve subjects, IMID patients showed a significant difference in the levels of anti-S-RBD IgG than HCW (− 33% at T2, after the full vaccination course), whereas no significant difference was observed at T3, 5 months after vaccination.

Initial immunogenicity data available for at-risk subgroups showed a high proportion of non-responders and low antibody responses to the SARS-CoV-2 spike protein in patients who received solid-organ transplantation [18], patients treated for solid cancer [19], or in patients with ongoing hemodialysis [20]. In contrast, the available studies to date show that a full course of mRNA vaccine in patients with IMID elicit a response in 78–100% of subjects [21], showing reduced serum antibody IgG titers and serum neutralizing activity as compared to healthy controls [10, 13, 14, 22–24]. Reduced humoral and cellular immune response (CD8 +) to COVID-19 mRNA vaccines were found by Haberman et al. in IMID treated with methotrexate and by Mahil et al. in psoriasis (47% and 62% responders, respectively) [23, 25], although this finding has not been confirmed in our and other studies (> 80% responders) [14, 21, 22, 26]. Some factors such as the characteristics of enrolled populations and selection criteria may explain this difference.

Even with limitations due to different assays and thresholds used across the available studies, the vaccine-elicited responses are largely indicative of a considerable immunogenicity. The results of the present study are in-line with those findings, and highlight that IMID treatments including GC, DMARDs, biologicals different from B-cell depleting agents could maintain IgG anti-S-RBD response until 5 months.

Of note, a strong correlation exists between serum neutralizing activity to SARS-COV-2 using PRNT_50_ and anti-S-RBD titer evaluated by the assay used in our study [16]. The median anti-S-RBD found in this study in IMID, respectively, 146.39 AU/mL and 60.65 AU/ml at T2 and T3, may correlate to a high neutralizing titer (≥ 1:160 PRNT_50_) for the majority of patients, and almost all those with > 50 AU/ml may have neutralizing titers of 1:40–1:80 up to 5 months after vaccination. This is in-line with predictive models of protection from COVID-19 infection and severe disease based on different COVID-19 vaccine trials [27]. Besides IMID, healthy subjects may show low humoral (spike antibody titer or neutralizing antibodies) or cellular response to vaccines. Anti-spike antibody titer (and the related neutralizing activity) declines over months in most vaccinated subjects [28, 29], with different rate between subjects previously positive and those naïve to SARS-CoV-2. However, our data do not show significant differences at 5 months between IMID and HCW. The main difference might be in the peak IgG titer reached after vaccine booster, then this difference decreases over a few months due to a faster decrease in healthy subjects. (− 79% than T2 peak among HCW vs. − 59% among IMID). We are not aware of data showing a different efficacy (excess of moderate/severe illness or symptomatic disease) in the first 5–6 months after mRNA vaccination with two doses in IMID versus healthy subjects [30].

Age had an influence at T1 and T2, with a stronger negative correlation in IMID patients than HCW, but not at T3. Previous reports also found that the age impact was time-related [28, 31], and may be linked to an IgG response that is faster and of higher magnitude in younger subjects for a short period after the two vaccine doses also for IMID.

In IMID patients, with ongoing immunosuppressive regimens also based on drug combinations and no drug suspension before or during the vaccination schedule, we found no differences in rates of responders neither in anti-S-RBD titer and this may be useful to study the most appropriate vaccination strategies for these patients. Simpler vaccination schedules may increase adherence of patients. The magnitude of response impairment to mRNA COVID-19 vaccine in patients with ongoing B-cells depleting therapies suggest that precise strategies of vaccination should be implemented in the next future [11, 14, 17]. Strategies to rescue the immunogenicity in these patients by repeating a full vaccine course [32] or giving a third dose [33] might be explored in larger studies. Peripheral lymphocytes subpopulations (especially CD19 + counts > 27 cells/μL) are predictive of vaccine response and there is a short period observed from vaccine to infection [34, 35], that may indicate the importance of detecting primary non-responders. These findings preliminary suggest that patients treated with anti-CD20 may deserve attention on B-cell reconstitution and/or antibody testing and strict adherence to personal protective measures.

We also assessed humoral responses of nine IMID individuals receiving immunosuppression who had previous natural SARS-CoV-2 infection and received two vaccine doses. There was evidence of robust IgG anti-RBD response in all but one patient, and the humoral response exceeded that of our SARS-CoV-2-naive participants and was similar to that of HCW with previous infection. The non-responder subject underwent her last rituximab course and COVID-19, respectively, 14 and 12 months before vaccination and peripheral B-cell depletion persisted at time of vaccination. Similar findings have been reported [22, 23].

Systemic side effects after vaccination were less frequent in IMID than in controls as in many previous studies [13, 14, 23], this finding being significantly associated with current treatment with glucocorticoids, but the impact of other factors and biases should be better addressed.

The study limitations are the number of recruited subjects in some subgroups and the absence of formal studies with neutralization assays or cellular immunity, as well as the absence of adenoviral-vector-based vaccinated subjects.

This data highlight the immunogenicity and importance of COVID-19 vaccination in patients with IMID as a measure to contrast pandemics, taking into consideration specific groups such as those treated with B-cell depleting agents. Epidemiological and efficacy studies for special groups are needed to understand whether there is a different immune response kinetics to mRNA vaccination, differentiate the risk for specific subgroups in order to tailor vaccination campaign and determine if further vaccine doses are warranted and their optimal timing.

## Supplementary Information

Below is the link to the electronic supplementary material.

Supplementary file1 (DOCX 43 KB)

## Data Availability

Data and materials are available from corresponding authors on reasonable request.

## Abbreviations

CLIA: Chemiluminescent analytical system
COVID-19: Coronavirus disease 2019
csDMARDs: Conventional synthetic and targeted synthetic DMARDs
IMID: Immune-mediated inflammatory diseases
HCW: Healthy healthcare workers
PRNT: Plaque reduction neutralization test
RBD: Receptor binding domain
SARS-CoV-2: Severe acute respiratory syndrome coronavirus 2
