# Supplementary material for: Evaluation of antibody response to BNT162b2 mRNA COVID-19 vaccine in patients affected by immune-mediated inflammatory diseases up to 5 months after vaccination
Source: Clin Exp Med. 2021 Nov 5;22(3):477–85. doi: 10.1007/s10238-021-00771-3 (PMC8570239; doi:10.1007/s10238-021-00771-3)
Supplement: Supplementary file 1 — Supplementary file1 (DOCX 43 KB) [file 10238_2021_771_MOESM1_ESM.docx]

Supplementary material for:

**Evaluation of antibody response to BNT162b2 mRNA COVID-19 vaccine in patients affected by immune-mediated inflammatory diseases up to 5 months after vaccination**

Davide Firinu^a^, Andrea Perra^b,c^, Marcello Campagna^a^, Roberto Littera^c,d^, Giuseppe Fenu^e^, Federico Meloni^a^, Selene Cipri^d^, Francesca Sedda^b^, Maria Conti^a^, Michela Miglianti^a^, Giulia Costanzo^a^, Marta Secci^a^, Gianmario Usai^a^, Mauro Giovanni Carta^a^, Riccardo Cappai^f^, Germano Orrù^a^, Stefano Del Giacco^a^, Ferdinando Coghe^f^, Luchino Chessa^a,c^

^a^ Department of Medical Sciences and Public Health, University of Cagliari, Cagliari, Italy

^b^ Department of Biomedical Sciences, University of Cagliari, Cagliari, Italy

^c^ Associazione per l’Avanzamento della Ricerca per i Trapianti O.d.V., non profit organisation,

Cagliari, Italy

^d^ Medical Genetics, Department of Medical Sciences and Public Health, University of Cagliari, Cagliari, Italy

^e^ Multiple Sclerosis Center, Binaghi Hospital, ATS Sardegna, Department of Medical Sciences and Public Health, University of Cagliari, Italy.

^f^ Laboratory Clinical Chemical Analysis and Microbiology, University Hospital of Cagliari, Italy.

**Corresponding author**:

Dr. Davide Firinu,

Department of Medical Sciences and Public Health, University of Cagliari.

and Unit of Internal Medicine, Policlinico Universitario – AOU di Cagliari

Postal address: Azienda Ospedaliero Universitaria, SS 554-Bivio Sestu, 09042 Monserrato (CA), Italy.

Telephone: +39 070 51096128 - Fax +39 070 51096227.

E-mail: davide.firinu@unica.it

**Supplementary table S1**

**SARS-CoV-2 Anti-S-RBD IgG humoral response in naïve IMID patients**

Supplementary table S1 legend: IgG titer Anti-S-RBD IgG (AU/L) elicited by BNT162b2 at T1 (booster dose), T2 (28 days after booster), and T3 (151 days after first dose) as determined by CLIA among IMIDs subgroups, naïve to SARS-CoV-2 infection.

|  | | | | | |
| --- | --- | --- | --- | --- | --- |
|  | | | SARS-CoV-2  anti-RBD IgG  T1 | SARS-CoV-2  anti-RBD IgG  T2 | SARS-CoV-2  anti-RBD IgG  T3 |
| SLE | n |  | 5 | 17 | 4 |
|  | Median | | 17.54 | 201.30 | 79.44 |
|  | Quartile | 25 | 8.15 | 44.50 | 33.38 |
|  |  | 50 | 17.54 | 201.30 | 79.44 |
|  |  | 75 | 24.85 | 330.45 | 138.06 |
| RA | n |  | 5 | 16 | 4 |
|  | Median | | 12.11 | 93.73 | 31.36 |
|  | Quartile | 25 | 4.50 | 44.97 | 17.89 |
|  |  | 50 | 12.11 | 93.73 | 31.36 |
|  |  | 75 | 43.07 | 347.12 | 69.41 |
| PsA/Psoriasis/AS | n |  | 10 | 17 | 8 |
|  | Median | | 14.42 | 184.60 | 58.32 |
|  | Quartile | 25 | 9.36 | 96.73 | 20.99 |
|  |  | 50 | 14.42 | 184.60 | 58.32 |
|  |  | 75 | 34.24 | 290.05 | 116.10 |
| IBD | n |  | 1 | 3 | 1 |
|  | Median | | 246.20 | 212.80 | 234.42 |
|  | Quartile | 25 | 246.20 | 117.90 | 234.42 |
|  |  | 50 | 246.20 | 212.80 | 234.42 |
|  |  | 75 | 246.20 | . | 234.42 |
| Miscellaneous systemic disorders | n |  | 4 | 13 | 3 |
|  | Median | | 5.45 | 80.12 | 90.49 |
|  | Quartile | 25 | 4.23 | 45.84 | 33.31 |
|  |  | 50 | 5.45 | 80.12 | 90.49 |
|  |  | 75 | 26.24 | 263.35 | . |

**Supplementary Table S2. Anti-S-RBD IgG titer of IMID patients, naïve for SARS-CoV-2, responders to BNT162b2 vaccine, according to ongoing treatment with csDMARD**

Supplementary table S2 legend: IgG titer Anti-S-RBD IgG (AU/L) elicited by BNT162b2 at T1 (booster dose), T2 (28 days after booster), and T3 (151 days after first dose) as determined by CLIA among IMIDs subjects naïve to SARS-CoV-2 infection.

csDMARDs, conventional synthetic and targeted synthetic DMARDs included: csDMARDs: methotrexate, sulfasalazine, leflunomide, hydroxychloroquine, mycophenolate, azathioprine, cyclosporin

| csDMARDs | | n | Percentile | | |
| --- | --- | --- | --- | --- | --- |
|  |  |  | 25th | 50th (median) | 75th |
| No | IgG anti-S-RBD T1 | 17 | 6.75 | 14.72 | 34.62 |
|  | IgG anti-S-RBD T2 | 33 | 58.31 | 139.70 | 243.60 |
|  | IgG anti-S-RBD T3 | 14 | 20.96 | 58.32 | 113.75 |
| Yes | IgG anti-S-RBD T1 | 8 | 2.13 | 12.50 | 19.88 |
|  | IgG anti-S-RBD T2 | 33 | 55.30 | 170.30 | 323.90 |
|  | IgG anti-S-RBD T3 | 6 | 34.07 | 75.26 | 104.042 |

| **Supplementary Table S3. Anti-S-RBD IgG titer of IMID patients, naïve for SARS-CoV-2, responders to BNT162b2 vaccine, according to treatment with anti-CD20 agents.**  Supplementary table S3 legend: IgG titer Anti-S-RBD IgG (AU/L) elicited by BNT162b2 at T1 (booster dose), T2 (28 days after booster), and T3 (151 days after first dose) as determined by CLIA among IMIDs subjects previously treated with anti-CD20, naïve to SARS-CoV-2 infection. | | | | | |
| --- | --- | --- | --- | --- | --- |
| anti-CD20 | | | IgG anti-S-RBD  T1 | IgG anti-S-RBD  T2 | IgG anti-S-RBD  T3 |
| No | n |  | 32 | 71 | 23 |
|  | Median | | 13.82 | 137.80 | 60.65 |
|  | Percentile | 25 | 4.38 | 48.24 | 21.10 |
|  |  | 50 | 13.82 | 137.80 | 60.65 |
|  |  | 75 | 31.08 | 270.00 | 110.80 |
| Yes | n |  | 3 | 6 | 2 |
|  | Median | | .81 | 2.13 | 1.76 |
|  | Percentile | 25 | .51 | .77 | .53 |
|  |  | 50 | .81 | 2.13 | 1.76 |
|  |  | 75 | . | 81.95 | . |

Supplementary table S4.

**Correlation of age and antibody response to vaccination at T1, T2 and T3 among healthy HCW.**

|  | | | IgG anti-S-RBD  T1 | IgG anti-S-RBD  T2 | IgG anti-S-RBD  T3 |
| --- | --- | --- | --- | --- | --- |
| Spearman's rho | Age (years) | Correlation Coefficient | -.244^**^ | -.116^**^ | -.100 |
|  |  | Sig. (1-tailed) | .000 | .007 | .181 |
|  |  | N | 365 | 453 | 86 |
| **. Correlation is significant at the 0.01 level (1-tailed). | | | | | |
| *. Correlation is significant at the 0.05 level (1-tailed). | | | | | |

Supplementary table S5.

**Correlation of age and antibody response to vaccination at T1, T2 and T3 among IMID.**

|  | | | IgG anti-S-RBD  T1 | IgG anti-S-RBD  T2 | IgG anti-S-RBD  T3 |
| --- | --- | --- | --- | --- | --- |
| Spearman's rho | Age (years) | Correlation Coefficient | -.410^**^ | -.256^*^ | .018 |
|  |  | Sig. (1-tailed) | .002 | .027 | .479 |
|  |  | N | 47 | 57 | 11 |
| **. Correlation is significant at the 0.01 level (1-tailed). | | | | | |
| *. Correlation is significant at the 0.05 level (1-tailed). | | | | | |

Supplementary table S6.

**Linear regression model of age, gender and immunosuppressant and/or corticosteroid and antibody response to vaccination at T1, T2 and T3 among IMID.**

| 1. Dependent Variable: IgG CoV-2 RBD IgG T1 | | | | | | | | |
| --- | --- | --- | --- | --- | --- | --- | --- | --- |
| Model | | Unstandardized Coefficients | | Standardized Coefficients | t | Sig. | 95.0% Confidence Interval for B | |
|  |  | B | Std. Error | Beta |  |  | Lower Bound | Upper Bound |
| 1 | (Constant) | 617.890 | 211.102 |  | 2.927 | .006 | 190.537 | 1045.243 |
|  | age | -7.463 | 3.376 | -.336 | -2.211 | .033 | -14.298 | -.628 |
|  | gender | -118.384 | 112.990 | -.160 | -1.048 | .301 | -347.121 | 110.353 |
|  | drugs | -18.377 | 93.472 | -.030 | -.197 | .845 | -207.601 | 170.847 |
| 1. Dependent Variable: IgG CoV-2 RBD IgG T2 | | | | | | | | |
| Model | | Unstandardized Coefficients | | Standardized Coefficients | t | Sig. | 95.0% Confidence Interval for B | |
|  |  | B | Std. Error | Beta |  |  | Lower Bound | Upper Bound |
| 1 | (Constant) | 459.416 | 112.158 |  | 4.096 | .000 | 236.126 | 682.705 |
|  | age | -3.776 | 1.849 | -.227 | -2.042 | .045 | -7.457 | -.095 |
|  | gender | -47.363 | 56.977 | -.091 | -.831 | .408 | -160.796 | 66.070 |
|  | drugs | -27.926 | 60.338 | -.052 | -.463 | .645 | -148.050 | 92.197 |
| 1. Dependent Variable: IgG CoV-2 RBD IgG T3 | | | | | | | | |
| Model | | Unstandardized Coefficients | | Standardized Coefficients | t | Sig. | 95.0% Confidence Interval for B | |
|  |  | B | Std. Error | Beta |  |  | Lower Bound | Upper Bound |
| 1 | (Constant) | 259.066 | 73.008 |  | 3.548 | .002 | 108.036 | 410.095 |
|  | age | -2.881 | 1.313 | -.406 | -2.195 | .039 | -5.596 | -.165 |
|  | gender | -49.201 | 44.956 | -.221 | -1.094 | .285 | -142.199 | 43.797 |
|  | drugs | 6.389 | 31.959 | .040 | .200 | .843 | -59.723 | 72.501 |

Supplementary table S7. **Linear regression model of age, gender and immunosuppressant and/or corticosteroid, disease duration and antibody response to vaccination at T1, T2 and T3 among IMID.**

| 1. Dependent Variable: IgG CoV-2 RBD IgG T1 | | | | | | | | |
| --- | --- | --- | --- | --- | --- | --- | --- | --- |
| Model | | Unstandardized Coefficients | | Standardized Coefficients | t | Sig. | 95.0% Confidence Interval for B | |
|  |  | B | Std. Error | Beta |  |  | Lower Bound | Upper Bound |
| 1 | (Constant) | 211.72 | 236.40 |  | .896 | .382 | -284.93 | 708.38 |
|  | age | \| -1.249 \| \| --- \| | 3.784 | -.074 | -.330 | .745 | -9.19 | 6.70 |
|  | gender | -237.92 | 128.1 | -.446 | -1.86 | .080 | -507.03 | 31.18 |
|  | drugs | 115.12 | 109.56 | .223 | 1.051 | .307 | -115.04 | 345.29 |
|  | disease duration | 4.417 | 9.072 | .121 | .487 | .632 | -14.64 | 23.47 |
| 1. Dependent Variable: IgG CoV-2 RBD IgG T2 | | | | | | | | |
| Model | | Unstandardized Coefficients | | Standardized Coefficients | t | Sig. | 95.0% Confidence Interval for B | |
|  |  | B | Std. Error | Beta |  |  | Lower Bound | Upper Bound |
| 1 | (Constant) | 493.93 | 157.63 |  | 3,13 | .003 | 178.15 | 809.70 |
|  | age | -4.107 | 2,42 | -.222 | -1.69 | .096 | -8.96 | .755 |
|  | gender | -62.97 | 72.49 | -.116 | -.869 | .389 | -208.19 | 82.24 |
|  | drugs | -8.64 | 89.27 | -.013 | -.097 | .923 | -187.48 | 170.18 |
|  | disease duration | -.693 | 4.853 | -.019 | -.143 | .887 | -10.41 | 9.02 |
| 1. Dependent Variable: IgG CoV-2 RBD IgG T3 | | | | | | | | |
| Model | | Unstandardized Coefficients | | Standardized Coefficients | t | Sig. | 95.0% Confidence Interval for B | |
|  |  | B | Std. Error | Beta |  |  | Lower Bound | Upper Bound |
| 1 | (Constant) | 110.145 | 85.44 |  | 1.289 | .238 | -91.892 | 312.18 |
|  | age | -1.124 | 1.68 | -.247 | -.668 | .526 | -5.103 | 2.85 |
|  | gender | -88.422 | 46.91 | -.835 | -1.885 | .101 | -199.34 | 22.50 |
|  | drugs | 21.935 | 32.46 | .236 | .676 | .521 | -54.84 | 98.71 |
|  | disease duration | 4.877 | 2.809 | .748 | 1.736 | .126 | -1.766 | 11.519 |

The collinearity diagnostic test showed: Condition Index 18.9 with variance proportion 0.97, supporting the collinearity between age and disease duration.
